# Treatment compliance and effectiveness of a cognitive behavioural intervention for low back pain: a complier average causal effect approach to the BeST data set

**DOI:** 10.1186/1471-2474-15-17

**Published:** 2014-01-14

**Authors:** Christopher R Knox, Ranjit Lall, Zara Hansen, Sarah E Lamb

**Affiliations:** 1 Warwick Clinical Trials Unit, Warwick Medical School, University of Warwick, Coventry CV7 1AL, UK; 2 Nuffield Department of Orthopaedics, Kadoorie Critical Care Research Centre, Rheumatology and Musculoskeletal Sciences, University of Oxford, Oxford OX3 9DU, UK

**Keywords:** Compliance, CACE analysis, Low back pain, Cognitive behaviour therapy

## Abstract

**Background:**

Group cognitive behavioural intervention (CBI) is effective in reducing low-back pain and disability in comparison to advice in primary care. The aim of this analysis was to investigate the impact of compliance on estimates of treatment effect and to identify factors associated with compliance.

**Methods:**

In this multicentre trial, 701 adults with troublesome sub-acute or chronic low-back pain were recruited from 56 general practices. Participants were randomised to advice (control n = 233) or advice plus CBI (n = 468). Compliance was specified a priori as attending a minimum of three group sessions and the individual assessment. We estimated the complier average causal effect (CACE) of treatment.

**Results:**

Comparison of the CACE estimate of the mean treatment difference to the intention-to-treat (ITT) estimate at 12 months showed a greater benefit of CBI amongst participants compliant with treatment on the Roland Morris Questionnaire (CACE: 1.6 points, 95% CI 0.51 to 2.74; ITT: 1.3 points, 95% CI 0.55 to 2.07), the Modified Von Korff disability score (CACE: 12.1 points, 95% CI 6.07 to 18.17; ITT: 8.6 points, 95% CI 4.58 to 12.64) and the Modified von Korff pain score (CACE: 10.4 points, 95% CI 4.64 to 16.10; ITT: 7.0 points, 95% CI 3.26 to 10.74). People who were non-compliant were younger and had higher pain scores at randomisation.

**Conclusions:**

Treatment compliance is important in the effectiveness of group CBI. Younger people and those with more pain are at greater risk of non-compliance.

**Trial registration:**

Current Controlled Trials ISRCTN54717854

## Background

A common problem in clinical trials, as well as clinical practice, is the failure of patients to fully comply with the allocated treatment. In trials of therapist-led intervention packages non-compliance occurs when individuals randomised to the intervention do not attend the number of sessions deemed sufficient for the intervention to deliver a benefit. In a conventionally used intention-to-treat (ITT) analysis all participants are analysed according to their treatment allocation even if they attend few or none of the therapy sessions. The intention-to-treat estimate provides an estimate for the effect of being offered the intervention when often our interest lies in the effect of receiving the treatment.

Complier-average causal effect (CACE) modelling is an analytic approach that provides a robust estimate of the treatment effect amongst compliant participants [1]. We specified a priori that compliance was likely to be an important contributor to the effect of group cognitive behavioural therapy, and designed a trial that was sufficiently pragmatic to allow estimation of these effects in a generalizable sample and range of settings [2]. In the absence of published guidance, we defined compliance as attendance at the assessment session plus three of the six group sessions as we hypothesised that this would enable the key components of the cognitive behavioural intervention to be delivered, although not necessarily re-enforced. In the original intention-to-treat analysis, we demonstrated that group cognitive therapy was effective at and beyond 12 months in a range of clinically relevant outcome measures, with standardised effect sizes mostly in the moderate range [3,4].

Here our aim was to investigate the nature and impact of non-compliance on the outcomes of group based cognitive behavioural intervention reported by Lamb et al. [3]. In non-technical terms, the concept of CACE is predicated as follows. At the outset of a trial, we assume that all participants have an unobservable characteristic (or set of characteristics, known as a latent variable) that determines whether they would comply or not with the test intervention. With randomisation, we assume that these characteristics are equally distributed across each arm of the trial, and that the proportion of “would be compliers with the test intervention” is therefore also equally distributed across the trial arms [5,6]. Only those randomised to the test arm have the opportunity to comply with the intervention and we are able to observe the proportion of compliers in the test arm. Because the proportion of would be compliers is equally distributed at the outset, we are able to estimate the proportion in the control group, from the proportion that are observed in the treatment group. We are also able to infer the unobserved mean of the non-compliers in the control group, from the observed average of the non-compliers in the treatment group. By representing the treatment difference observed through intention to treatment analysis as a product of the proportion of compliers and the complier averaged difference, and then re-organising the algebra, it is possible to estimate the complier averaged difference (CACE). Assumptions about missing data are important, and further refinement is added by inclusion of baseline co-variates through more sophisticated statistical techniques. An important assumption is that the offer of the intervention does not influence the outcome [7]. Non-compliers should, on average, have the same mean score in the intervention group as the control group [8,9].

## Methods

### Participants and procedures

The Back Skills Training (BeST) Trial was a multicentre randomised controlled trial and the design, intervention, and main analyses have been reported in detail elsewhere [2,3,10,11]. In short, 701 participants over 18 years of age were recruited from primary care with at least moderately troublesome nonspecific low back pain (LBP) present for greater than 6 weeks. Participants were randomised, in a ratio of 1:2 respectively, to advice alone (control) or advice plus cognitive behavioural intervention.

All participants received a 15-minute session of active management advice, which included advice on remaining active, avoidance of bed rest, appropriate use of pain medication and symptom management, supplemented by a copy of *The Back Book*[12].

In addition, participants in the intervention group attended the Back Skills Training (BeST) programme, consisting of an individual assessment (up to 1.5 hours duration) and six sessions of group therapy (1.5 hours duration each) using a cognitive–behavioural approach. We aimed for a group size of about 8 participants facilitated by one therapist. We trained 19 therapists (14 Physiotherapists, 2 Occupational Therapists, 2 psychologists and 1 nurse) to deliver the programme over a 2-day training course. The intervention has been described in detail elsewhere [10]. Relevant to this paper is the content and sequence of material in each of the sessions. The assessment session aimed to gather information about the participant’s history of LBP and identify any unhelpful beliefs that the participant might have about their LBP. The assessment was also an opportunity to set three collaborative treatment goals, one of which we pre-specified as an exercise/physical activity goal because of the recognised importance of physical activity for managing low back pain. In addition the concepts of baseline setting and pacing were introduced during the assessment session. Thereafter the group sessions were structured around the themes shown in Table 1. Therapists were provided with a detailed manual describing all of the procedures for each session, and supporting materials. Each participant was provided with a summary at the end of each session, along with “homework” to practice before the next session, such as relaxation techniques. Those people not attending a session were mailed out the session summaries and homework. We recorded attendance of participants along with reasons for non-attendance, where provided.

**Table 1:** Details of the contents of the cognitive behavioural intervention group sessions

**Session number**	**1**	**2**	**3**	**4**	**5**	**6**
***Assessment***						
History taking including current problems and eliciting beliefs on LBP and activity						
Collaborative goal setting with plan to start activity goal						
Exercises chosen collaboratively from options with level negotiated						
Exercises practised and progression discussed						
***Understanding pain***	✓					
Group activity to demonstrate hurt does not equal harm						
Current thinking on causes of long-term pain explained						
Discussion on groups experience of alternative treatments for LBP with reference to research evidence and need to self manage						
***Benefits of exercise***	✓					
Discussion of physical impact of inactivity or altered activity and how changes impact on pain (disuse syndrome)						
Discussion on effects of activity/exercise						
Introduction to LBP model						
***Pain fluctuations***		✓				
Overactivity/underactivity cycle explained						
Use of pacing						
Group problem solving for a specific task that tends to be ‘overdone’ e.g. gardening						
***Working out starting point for exercises or activities***		✓				
How to use baseline setting						
***How to set goals***		✓				
SMART system used to break down an example goal						
Feedback from group on how progressing with goals from assessment						
Group problem-solving problems with goals						
***Unhelpful thoughts and feelings***			✓			
Styles of unhelpful thinking discussed, including catastrophising						
Link with unhelpful behaviours						
Identifying unhelpful thoughts						
Group problem-solving for challenging unhelpful thoughts						
***Restarting activities or hobbies***				✓		
Discussion on activities commonly avoided in LBP						
Fear avoidance cycle						
Group problem-solving out of cycle						
Development of specific goals relating to restarting activities						
***When pain worries us***					✓	
Effect of attention to pain explored through group activity						
Hypervigilance cycle used to link unhelpful thoughts and behaviours						
Group problem-solving out of cycle						
Discussion on the use of medication/distraction/alternating activities						
***Coping with flare-ups***						✓
Discussion on causes of flare-ups						
Plan of what to do in and out of flare-ups						
Revision of topics over previous sessions and questions						

LBP, lower back pain.

### Outcome measurements

Data were collected by postal questionnaire at 3, 6 and 12 months after randomisation. The primary outcomes were the Roland Morris Disability Questionnaire (RMDQ; scale 0–24, where lower scores indicate less severe disability) [13] and modified Von Korff (MVK) scales of pain and disability (scale 0–100%, where lower scores indicate less pain and disability) [14]. Generic health-related quality of life was collected using the EQ-5D (scale -0.594 to 1, where lower scores indicate poorer quality of life) [15]. Telephone follow-up was attempted for the modified Von Korff scale in cases of non-response to the postal questionnaire.

### Ethics

The West Midlands Multi-centre Research Ethics Committee approved the trial protocol and longer-term follow-up (MREC/03/7/04). All participants gave written informed consent.

### Definition of compliance

Compliance was defined a priori as attendance at the initial assessment and at least three subsequent sessions. The cognitive behavioural intervention was only available to participants randomly allocated to receive it and so compliance status is only observable in the intervention group. Compliance was observed at the intervention stage of the study. Loss to follow up is still used to refer to patients for whom it was not possible to collect outcome data at the follow-up time points.

### Statistical analysis

We examined the baseline characteristics of participants randomised to the CBI arm, by compliance status. In addition, we report the baseline characteristics of participants randomised to the control arm.

The effect of treatment was estimated as the mean change scores from baseline as this was the only method that remedied substantial skew in the data, and was consistent with the original analysis [2,3]. We produced summary estimates of the treatment effect for compliers and non-compliers in the intervention arm using linear regression analysis adjusted for baseline covariates as specified in the original analysis. This included adjustment for the baseline value of the change score.

We produced CACE estimates of the difference between the mean score for the compliers in the intervention group compared to the would-be compliers in the control group [16]. To obtain a CACE estimate we used a latent class model approach using the gllamm command in STATA [9,17,18]. This CACE model estimates the compliers in the control group and compares these to the observed compliers in the intervention group to estimate a treatment effect amongst compliers. The amount of unobserved compliers in the control group can be estimated using the proportion of compliers in the intervention group based on the assumption that the proportion of compliers is balanced across the treatment arms [5,6]. All participants were analysed according to their treatment allocation.

A CACE model including baseline covariates associated with the two responses, compliance for the intervention arm and outcome for the whole sample, was fitted and compared to a null model using the likelihood ratio test. The compliance part of the model was adjusted for age and baseline modified Von Korff score as these were found to be associated with compliance. The outcome part of the model is adjusted for the baseline covariates of age, gender, severity of back pain, centre and baseline values of the outcome score as specified in the original BeST analysis [3].

The analysis includes all eligible randomised participants who provided follow-up data. Our CACE analysis assumes that whether or not a participant provides follow-up data is determined by their compliance status and so missing data is ignorable [8,19]. Sensitivity to missing data was investigated using a multiple imputation analysis. Estimates from the CACE model were compared to ITT estimates from an adjusted linear regression with participants analysed according to allocation assignment regardless of compliance. We provide a qualitative comparison of the CACE and ITT models using the standardised effect size (calculated as the unadjusted mean difference between the groups divided by the pooled standard deviation at baseline). The CACE analysis was repeated with compliance re-defined as attendance at the individual assessment, attendance at the individual assessment and one or more group therapy sessions and two or more group therapy sessions. A range of outcomes were analysed at 3, 6 and 12 months and all analyses were carried out using STATA version 11 [18].

## Results

### Participants

Of the 701 randomised participants 598 (85%) provided 12-month follow up data (advice alone n = 199/233 (85%); advice plus cognitive behavioural intervention, n = 399/468 (85%)). For the outcome measures used in the CACE analyses the number of contributing participants ranged from 498 with complete Roland Morris questionnaire scores at 12 months to 587 with complete modified Von Korff pain scores and EQ-5D scores at 12 months, with no difference in the levels of missing data between treatment groups. Due to missing data in the outcome and predictors the number of participants used in the analyses differs for each month and outcome score. The number of participants in the ITT analysis is the same is in the CACE analysis for each month and outcome score. There was one participant randomised to the advice group who received the cognitive behavioural intervention. They were analysed as intention to treat.

**Figure 1:**
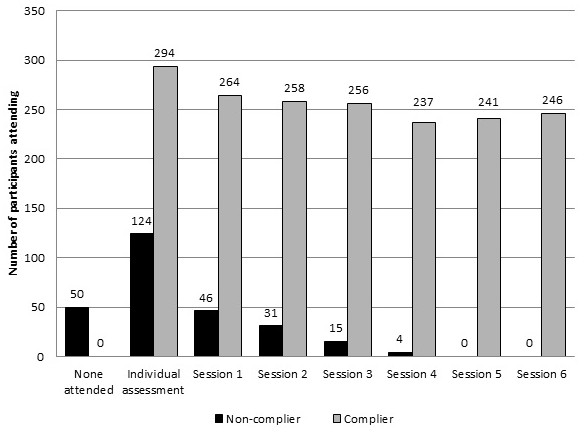
**Attendance at cognitive behavioural intervention sessions by compliers and non-compliers.** Number of compliers (n = 294) and non-compliers (n = 174) in the CBI group (n = 468) attending each cognitive behavioural intervention session with a maximum of six sessions plus the initial individual assessment.

Levels of attendance at the cognitive behavioural assessment and group sessions are shown in Figure 1. Of the 468 participants assigned to cognitive behavioural intervention, 174 (37%) did not achieve the compliance threshold. For the non-compliant group, 50/174 (29%) people did not attend the assessment or sessions, and 59/174 (34%) attended the assessment only. The remainder of people who were non-compliant (65/174) attended the assessment and an average of 1.5 (SD 0.50) sessions. The average number of sessions attended by the compliant group was 5.1 (SD 0.91).

We obtained reasons for non-attendance from 78 (45%) of the non-compliers. The primary reasons given for not attending were feeling unwell (18/78 (10%)), changes to work (15/78 (9%)) and family issues (14/78 (8%)). The attainment of the compliance threshold was broadly similar across the different centres (22/38 (58%) to 84/127 (66%)), as were patterns of missing data. The number of patients reaching the compliance threshold varied across the 19 therapists (3/8 (38%) to 4/5 (80%)). However, there was less deviation in the complier numbers where there were more than 10 patients per therapist (8/15 (53%) to 13/18 (72%)).

Loss to follow up was significantly associated with compliance. At 12 months 113 out of 174 (65%) non-compliers provided a modified Von Korff pain score, compared with 279 out of 294 (95%) compliers (chi squared p-value < 0.001). On the Roland Morris questionnaire 72 out of 174 (41%) non-compliers provided complete data, compared to 267 out of 294 (91%) compliers (chi squared p-value < 0.001).

### Complier characteristics

Table 2 shows the baseline characteristics of the compliers and non-compliers in the intervention group. Noticeably, in comparison to non-compliers, compliers were older (mean difference 4.5 years, 95% CI 1.8-7.2), had a longer duration of back pain (mean difference 2.8 years, 95% CI 0.2-5.3), had less frequent back pain (odds ratio 0.5, 95% CI 0.3-0.8) and had lower baseline modified Von Korff pain scores (mean difference 4.6 points, 95% CI 0.9-8.2). In multi-variate analysis, two baseline factors emerged as being associated with compliance. These were age (odds ratio 1.02, 95% CI 1.01-1.04), and a worse modified Von Korff pain score (odds ratio 0.99, 95% CI 0.98-1.00).

**Table 2:** Baseline characteristics of randomised participants by compliance status; complier, non-complier or control

	**Advice plus cognitive behavioural intervention (n = 468)**				
	**Control**	**Complier**	**Non-complier**	Mean difference^*^	**p value**^**†**^
	**(n = 233)**	**(n = 294)**	**(n = 174)**	**(95% CI)**	
Age (years)	54.1 (14.9)	55.0 (14.3)	50.5 (14.8)	4.5 (1.8, 7.2)	0.001
Sex				-	0.694
Female	142 (60.9%)	173 (58.8%)	105 (60.3%)		
Male	90 (38.6%)	121 (41.2%)	68 (39.1%)		
Missing	1 (0.4%)	0	1 (0.6%)		
Ethnic origin				-	0.258
White	206 (88.4%)	268 (91.2%)	144 (82.8%)		
Mixed	3 (1.3%)	2 (0.7%)	2 (1.1%)		
Asian	8 (3.4%)	9 (3.1%)	12 (6.9%)		
Black	4 (1.7%)	4 (1.4%)	3 (1.7%)		
Chinese	1 (0.4%)	1 (0.3%)	0		
Missing	11 (4.7%)	10 (3.4%)	13 (7.5%)		
Severity of back pain				-	0.802
Moderately troublesome	130 (55.8%)	157 (53.4%)	95 (54.6%)		
Very or extremely troublesome	103 (44.2%)	137 (46.6%)	79 (45.4%)		
Age left full-time education (years)				-	0.584
≤16	121 (51.9%)	168 (57.1%)	98 (56.3%)		
17-19	61 (26.2%)	65 (22.1%)	39 (22.4%)		
≥20	49 (17.2%)	48 (16.3%)	25 (14.4%)		
Still in full-time education	2 (0.9%)	0	1 (0.6%)		
Missing	9 (3.9%)	13 (4.4%)	11 (6.3%)		
In employment†				-	0.612
Yes	110 (47.2%)	147 (50.0%)	91 (52.3%)		
No	122 (52.4%)	146 (49.7%)	82 (47.1%)		
Retired	77 (63.1%)	94 (64.4%)	36 (43.9%)		
At home not looking for work	4 (3.3%)	12 (8.2%)	13 (15.9%)		
Unable to work because of back pain	11 (9.0%)	12 (8.2%)	10 (12.2%)		
Unable to work because of other illness	9 (7.4%)	10 (6.8%)	6 (7.3%)		
Unemployed and looking for work	8 (6.6%)	1 (0.7%)	3 (3.7%)		
In full-time education	2 (1.6%)	0	0		
Other	3 (2.5%)	1 (0.7%)	5 (6.1%)		
Reason not given	8 (6.6%)	16 (11.0%)	9 (11.0%)		
Duration of back pain (years since first onset)	13.2 (12.7)	14.1 (14.2)	11.4 (11.2)	2.8 (0.2, 5.3)	0.034
Frequency of back pain (past 6 weeks)				-	0.009
Everyday	162 (69.5%)	189 (64.3%)	126 (72.4%)		
Less often than everyday	47 (20.2%)	82 (27.9%)	29 (16.7%)		
Missing	24 (10.3%)	23 (7.8%)	19 (10.9%)		
Roland Morris questionnaire	8.5 (4.7)	8.6 (4.9)	9.1 (5.2)	-0.4 (-1.4, 0.5)	0.357
Modified Von Korff disability	46.2 (23.8)	47.0 (23.6)	51.0 (24.1)	-4.0 (-8.6, 0.5)	0.083
Modified Von Korff pain	59.4 (19.5)	57.6 (19.3)	62.2 (18.9)	-4.6 (-8.2, -1.0)	0.014
EQ-5D	0.6 (0.3)	0.6 (0.3)	0.5 (0.3)	0.1 (-0.01, 0.1)	0.082

Data are mean (SD) or n (%). Participants in the control group were assigned to receive advice only.

*Mean difference between compliers and non-compliers in cognitive behavioural intervention arm.

†Reported p values are from t test where mean differences are reported and chi-squared tests elsewhere.

Adjusted estimates of the mean scores on the Roland Morris questionnaire, modified Von Korff score and EQ-5D are reported by intervention arm and by compliance status in Table 3. The effect of compliance was most evident on the pain and disability outcomes at the time points closest to the intervention delivery, where compliers experienced at least a doubling of response in comparison to non-compliers. By the 12 month follow up, non-compliers had recovered to a similar level as the compliers, the only exceptions being the Modified Von Korff Disability score at 12 months, where compliers continued to report greater benefits from the cognitive behavioural intervention. Compliers experienced greater gains in EQ-5D scores at 3 months and these remained stable thereafter, whereas non-compliers reported a gradual improvement in EQ-5D, with no statistically significant difference by compliance status at either 6 or 12 months.

**Table 3:** ITT estimates of clinical effectiveness at 3, 6 and 12 months

	**All**		**Advice plus cognitive behavioural intervention**			
	**Control**	**Advice plus cognitive behavioural intervention**	**Non-complier**	**Complier**	Mean compliance difference (95% CI)^*^	**p value**
	Mean change from baseline (95% CI)^*^		Mean change from baseline (95% CI)^*^			
**Roland Morris Questionnaire (points)**^**†**^						
3 months	0.9 (0.32, 1.53)	2.0 (1.55, 2.46)	0.9 (-0.06, 1.83)	2.3 (1.82, 2.85)	1.5 (0.44, 2.47)	0.005
6 months	1.0 (0.38, 1.64)	2.5 (1.98, 2.92)	1.6 (0.68, 2.58)	2.7 (2.18, 3.21)	1.0 (-0.00, 2.07)	0.05
12 months	1.0 (0.36, 1.70)	2.3 (1.86, 2.82)	2.1 (1.12, 3.07)	2.5 (1.99, 3.09)	0.4 (-0.62, 1.52)	0.41
**Modified Von Korff disability (%)**^**†**^						
3 months	8.8 (5.42, 12.14)	13.4 (10.81, 16.03)	6.5 (1.27, 11.65)	14.6 (11.56, 17.71)	8.2 (2.51, 13.82)	0.005
6 months	5.7 (2.17, 9.19)	14.0 (11.43, 16.60)	9.0 (4.30, 13.67)	15.2 (12.07, 18.42)	6.3 (0.99, 11.53)	0.02
12 months	5.3 (1.82, 8.78)	13.9 (11.32, 16.50)	10.0 (5.48, 14.60)	16.0 (12.88, 19.12)	6.0 (0.78, 11.14)	0.024
**Modified Von Korff pain (%)**^**†**^						
3 months	5.4 (2.41, 8.30)	12.3 (10.02, 14.51)	7.4 (2.85, 11.87)	13.6 (10.94, 16.22)	6.2 (1.31, 11.12)	0.013
6 months	5.6 (2.27, 8.85)	13.8 (11.38, 16.18)	8.8 (4.44, 13.24)	15.5 (12.51, 18.41)	6.6 (1.67, 11.58)	0.009
12 months	6.4 (3.12, 9.62)	13.4 (10.98, 15.76)	11.6 (7.28, 15.85)	14.4 (11.52, 17.21)	2.8 (-2.05, 7.65)	0.257
**EQ-5D**						
3 months	0.5 (0.51, 0.58)	0.6 (0.59, 0.65)	0.6 (0.50, 0.61)	0.6 (0.59, 0.66)	0.1 (0.01, 0.13)	0.014
6 months	0.6 (0.54, 0.62)	0.6 (0.59, 0.65)	0.6 (0.55, 0.64)	0.6 (0.60, 0.66)	0.03 (-0.03, 0.09)	0.293
12 months	0.6 (0.54, 0.61)	0.6 (0.60, 0.66)	0.6 (0.56, 0.66)	0.6 (0.59, 0.66)	0.01 (-0.04, 0.07)	0.616

Estimates of disability, pain and health related quality of life at 3, 6 and 12 months for all participants (ITT) and for compliers and non-compliers in the advice plus cognitive behavioural intervention arm with the difference between compliers and non-compliers.

Participants in the control group were assigned to receive advice only.

*Based on a linear regression model adjusted for age, sex, severity of back pain, centre and baseline value.

†The number of participants used in the regression analyses differs for each month on each score; due to missing data in the outcome and predictors.

### Estimates of the CACE model

The ITT and CACE estimates of the treatment effect are reported in Table 4. Co-variate adjustment for the CACE model provided a statistically significant better fit for all models (Likelihood Test p < 0.001 for all models), and hence only these models are reported. In all CACE models, with the exception of the Roland Morris questionnaire, the estimate of the mean treatment difference was greater than from the ITT analysis. In nearly all comparisons, the lower bound of the 95% confidence interval was also greater.

**Table 4:** ITT and CACE model estimates of treatment difference at 3, 6 and 12 months

	**ITT covariate model**		**Standardised effect at 12 months†**	**CACE covariate model**		**Standardised effect at 12 months‡**
	Mean treatment difference (95% CI)^*^	**p value**		**Mean treatment difference (95% CI)†**	**p value**	
**Roland Morris Questionnaire (points)**						
3 months	1.1 (0.39, 1.77)	0.002	..	1.1 (0.04, 2.22)	0.042	..
6 months	1.4 (0.72, 2.16)	<0.001	..	1.9 (0.87, 2.98)	<0.001	..
12 months	1.3 (0.55, 2.07)	0.001	0.31	1.6 (0.51, 2.74)	0.004	0.43
**Modified Von Korff disability (%)**						
3 months	4.6 (0.75, 8.52)	0.029	..	5.4 (-0.38, 11.15)	0.067	..
6 months	8.3 (4.32, 12.35)	<0.001	..	11.1 (5.30, 16.83)	<0.001	..
12 months	8.6 (4.58, 12.64)	<0.001	0.42	12.1 (6.07, 18.17)	<0.001	0.60
**Modified Von Korff pain (%)**						
3 months	6.9 (3.53, 10.29)	<0.001	..	8.8 (3.84, 13.82)	0.001	..
6 months	8.2 (4.48, 11.97)	<0.001	..	11.2 (5.89, 16.61)	<0.001	..
12 months	7.00 (3.26, 10.74)	<0.001	0.37	10.4 (4.64, 16.10)	<0.001	0.60
**EQ-5D**						
3 months	0.08 (0.033, 0.117)	<0.001	..	0.09 (0.020, 0.161)	0.012	..
6 months	0.04 (-0.007, 0.080)	0.098	..	0.06 (-0.011, 0.121)	0.102	..
12 months	0.05 (0.010, 0.097)	0.015	0.13	0.07 (0.008, 0.141)	0.029	0.36

*Based on a linear regression model adjusted for age, sex, severity of back pain, centre and baseline value.

†Based on a latent class model with outcome adjusted for age, sex, severity of back pain, centre and baseline value and compliance adjusted for age and baseline modified Von Korff pain score.

‡Standardised effect size is the unadjusted mean difference (standardised mean difference) between the groups divided by the pooled SD at baseline.

The CACE estimate of the treatment difference between advice alone and advice plus cognitive behavioural therapy on the Roland Morris questionnaire score is 1.6 points (95% CI 0.51–2.74) at 12 months. The estimated treatment difference for compliers is 12.1 points (6.07-18.17) on the Von Korff disability score and 10.4 points (4.64-16.10) on the Von Korff pain score at 12 months. The estimated treatment difference at 12 months for compliers on the EQ-5D is 0.07 (0.01-0.14) points. At 12 months the standardised effect size is increased for all measures using the CACE analysis. Comparing the ITT estimate to the CACE estimate in Table 4, the estimate of the standardised effect size is increased from 0.31 to 0.43 on the Roland Morris questionnaire, from 0.42 to 0.60 on the Von Korff disability score and from 0.37 to 0.60 on the Von Korff pain score. There is a threefold increase in the estimate of the standardised effect size on the EQ-5D, from an ITT estimate of 0.13 to a CACE estimate of 0.36.

Estimates from CACE analyses redefining compliance as attendance at the individual assessment only, attendance at the individual assessment and one or more group therapy sessions and two or more group therapy sessions are reported in Table 5. Based on definitions of compliance with a minimum requirement as attendance at the individual assessment the estimate of the mean treatment difference is greater than from the ITT analysis for all outcomes at 6 and 12 months.

**Table 5:** CACE model estimates of treatment difference with re-defined compliance

	**Assessment only**			**1 or more group sessions**			**2 or more group sessions**		
	Mean treatment difference (95% CI)^*^	**p value**	**Standardised effect at 12 months‡**	Mean treatment difference (95% CI)^*^	**p value**	**Standardised effect at 12 months‡**	Mean treatment difference (95% CI)^*^	**p value**	**Standardised effect at 12 months‡**
**Roland Morris Questionnaire (points)**^**†**^									
3 months	1.1 (0.30, 1.86)	0.007	..	0.8 (-0.10, 1.72)	0.079	..	1.1 (0.07, 2.09)	0.035	..
6 months	1.4 (0.59, 2.18)	0.001	..	1.7 (0.80, 2.60)	<0.001	..	1.8 (0.78, 2.74)	<0.001	..
12 months	1.4 (0.61, 2.25)	0.001	0.34	1.5 (0.55, 2.43)	0.002	0.38	1.5 (0.48, 2.56)	0.004	0.40
**Modified Von Korff disability (%)**^**†**^									
3 months	4.9 (0.61, 9.14)	0.025	..	4.6 (-0.27, 9.43)	0.064	..	5.1 (-0.24, 10.38)	0.061	..
6 months	8.7 (4.34, 13.07)	<0.001	..	9.6 (4.71, 14.53)	<0.001	..	10.3 (4.97, 15.65)	<0.001	..
12 months	9.4 (4.93, 13.78)	<0.001	0.46	10.3 (5.19, 15.35)	<0.001	0.52	11.1 (5.57, 16.71)	<0.001	0.56
**Modified Von Korff pain (%)**^**†**^									
3 months	7.2 (3.53, 10.93)	<0.001	..	7.5 (3.37, 11.73)	<0.001	..	8.2 (3.57, 12.78)	<0.001	..
6 months	8.7 (4.59, 12.76)	<0.001	..	9.7 (5.10, 14.29)	<0.001	..	10.4 (5.43, 15.40)	<0.001	..
12 months	7.9 (3.69, 12.03)	<0.001	0.43	8.8 (3.99, 13.58)	<0.001	0.50	9.4 (4.08, 14.66)	0.001	0.53
**EQ-5D**^**†**^									
3 months	0.1 (0.01, 0.11)	0.015	..	0.1 (0.02, 0.14)	0.014	..	0.1 (0.01, 0.14)	0.024	..
6 months	0.04 (-0.01, 0.09)	0.12	..	0.04 (-0.01, 1.00)	0.128	..	0.1 (-0.01, 0.11)	0.102	..
12 months	0.1 (0.04, 0.13)	<0.001	0.40	0.1 (0.01, 0.12)	0.022	0.32	0.1 (0.01, 0.13)	0.023	0.36

CACE model estimates at 3, 6 and 12 months with compliance re-defined as attendance at the individual assessment only, attendance at the individual assessment and one or more group therapy sessions and two or more group therapy sessions.

^*^Based on a latent class model with outcome adjusted for age, sex, severity of back pain, centre and baseline value and compliance adjusted for age and baseline modified Von Korff pain score with the exception of modified Von Korff pain score at 6 months where compliance is only adjusted for age.

^†^The number of participants used in the regression analyses differs for each month on each score; due to missing data in the outcome and predictors.

‡Standardised effect size is the unadjusted mean difference (standardised mean difference) between the groups divided by the pooled SD at baseline.

CACE model estimates and ITT estimates based on imputed datasets show a small reduction in the magnitude of the treatment difference at 3, 6 and 12 months with the exception of the Roland Morris questionnaire at 3 months. The CACE estimates remain greater than the ITT estimates and conclusion of significance of the treatment effect remains the same for all analyses based on the imputed data.

## Discussion

Few, if any, trials of low back pain interventions have considered the treatment effect amongst compliers using robust analyses. Both the complier average causal effect and linear regression analyses support the concept that compliance has an important role determining the size of treatment effect. People who achieved the compliance threshold achieved a larger benefit in clinical and health related quality of life outcomes in the year of follow up in comparison to advice only, than those who are less compliant.

The estimates from the CACE analysis provide the most robust indication of the treatment effect amongst compliers, although there are some limitations in the analysis we present. First is that there is no accepted method of defining compliance to cognitive behavioural interventions. We based our analysis on the compliance definition set out in the protocol and have examined different thresholds in further analyses due to potential limitations of this definition in the analysis model. Our definition of attendance at the initial assessment and three or more sessions was based on a collective judgement of the intervention designers that this would provide the essential components of the programme. This is broadly in keeping with other reports in the literature [20]. In those defined as non-compliers, some may have received some elements of the cognitive behavioural intervention, and this could possibly lead to an underestimate of the treatment effect amongst compliers [21]. All participants of the trial received a brief, best practice active management advice session from a nurse or physiotherapist. In the intervention arm, 71% of those deemed non-compliant had attended some part of the BeST intervention, most commonly the assessment +/- the first or second group session, and had received the session summary materials for all sessions. Despite this we still observed a substantial and statistically significant difference in the treatment effect estimate.

We quantified compliance in terms of treatment attendance. CACE analysis assumes that compliance is a pre-randomisation characteristic and hence equally distributed across the treatment arms. Our data supports the hypothesis that the characteristic of being a complier is associated with a larger treatment benefit than being a non-complier. Whether compliance is potentially modifiable, and whether modifying the attendance behaviour of patients results in better outcomes is less certain.

There are several potential mechanisms by which compliance is associated with a larger treatment effect. First is reverse causality, i.e. that compliance is driven by early response to treatment and that as opposed to compliance being important to response, the early response engenders compliance. High pain at study entry (i.e. a pre-randomisation variable) was associated with non-compliance. We cannot rule in or out this hypothesis as we did not take measures of response on a weekly basis. Future studies could address this issue, and this would be important in understanding what drives compliance in the context of low back pain. The second potential mechanism is that greater attendance results in re-enforcement of the key components of the intervention. In designing the intervention some of the concepts were repeated across sessions, and the therapists were encouraged to revisit and draw on the experiences and skills mastered in prior sessions. Participants undertook regular review of their treatment goals, and progression toward them. Finally, it is possible that the later sessions contain elements that are substantially important to the effect, or that the increasing social contact across repeated visits has a therapeutic value.

We are not able to conclude from our results whether a shorter programme (3 or less sessions) would be as good as six sessions, as we did not test this explicitly.

An important assumption of the CACE analysis, the exclusion restriction, is that for non-compliers in the intervention arm there is no additional benefit gained from the offer of treatment compared to participants randomised to the advice group [7,9]. In this study participants that are randomised to the intervention are treated as non-compliers even if they might have attended one or two sessions as well as the individual assessment. We would expect that at least some of these participants would have received a partial benefit and so this could violate the exclusion restriction and introduce bias. We used a CACE model adjusted for covariates associated with outcome score and compliance which protects against bias if, as described above, there is violation of the exclusion restriction assumption [21]. It has also been shown that bias introduced by violation of the exclusion criteria is increased in the CACE estimate if the compliance rate is very low [21]. This was not the case; compliance within the group cognitive behavioural therapy was 63%.

Further CACE analyses show that when compliance is defined as attendance at a minimum of the individual assessment the treatment effect is still estimated to be greater than the ITT estimate at 12 months. This is not unexpected as, in a parallel qualitative study, a number of the participants of the intervention mentioned how valuable they found the assessment session independently of the group sessions [2]. The treatment effect estimates based on attendance at the individual assessment can be defined as the treatment effect of partial and complete compliers; it is the treatment effect excluding complete non-compliers. This provides an unbiased estimate of treatment effect and again demonstrates that the treatment effect estimated from an ITT analysis is an underestimate of the actual effect of treatment.

We have compared the estimates from the CACE model to ITT estimates so that it is comparable to our original reporting. An as-treated analysis is not reported as in the presence of non-compliance this method is considered to be misleading and so does not provide robust estimates [22].

Missing follow up data was shown to be related to compliance and so identification of non-compliers could be useful in managing the collection of follow up data. It also follows that if compliance rates can be increased through encouraging attendance at all sessions then this should lead to an increase in the completeness of follow up data. Under the missing at random assumption used in this CACE analysis the outcome response behaviour is permitted to differ between complier groups in the intervention arm [23]. There is also evidence from prior methodological work that CACE estimates produced from latent class modelling are insensitive to the missing data assumption used, missing at random or latent ignorability [24]. We undertook additional sensitivity analyses using multiply imputed datasets, and these suggest that the CACE results are insensitive to the missing data.

The latent class modelling approach used here to derive CACE estimates does not account for clustering effects such as therapist effects. It is possible to undertake analyses including clustering in a model accounting for non-compliance as demonstrated by B Jo, T Asparouhov and BO Muthén [25]. However, our original ITT analyses demonstrated therapist and group effects were negligible.

This analysis shows that the effectiveness of group cognitive behavioural intervention is increased in compliers. Whilst we cannot be sure that compliance is a modifiable trait, research from other areas suggest that it is, and issues such as family and work commitments in scheduling of sessions, or even in the mode of delivery (for example requiring attendance at a face to face meeting), may render better compliance [26]. We did not use specific behavioural techniques to encourage attendance at the sessions (i.e. attendance at sessions was not identified as a goal), and this approach may add to improved compliance.

There are examples in the literature of analysis of trials subject to non-compliance but these are mainly limited to social interventions and trials of psychological treatments [8,22,27]. This current paper is distinctive in providing a CACE analysis of a trial of sub-acute or chronic low-back pain. CACE estimates are potentially more clinically relevant than effect estimates derived from ITT analyses and give a better indication of the effect of receiving treatment.

## Conclusion

Exploration of compliance data and use of a CACE analysis can aid the interpretation of the primary results of randomised trials. CACE analysis provides a means of estimating the effect of the receipt of treatment in a randomised trial in which not all patients were compliant. Supplementing estimates from an ITT analysis with CACE estimates helps to provide a more complete picture of the pragmatic effects of an intervention that can support clinical decision making.

## Competing interest

The authors have no competing interest to declare.

## Authors’ contributions

SEL was the chief investigator and grant holder of the trial and had full access to all of the data in the study. The statistical analysis was undertaken by CRK under the guidance of RL and SEL. An initial draft of the manuscript was written by CK. ZH re-drafted parts of the manuscript and provided helpful advice on the final revision. All authors contributed to and approved the final draft.

## Pre-publication history

The pre-publication history for this paper can be accessed here:

http://www.biomedcentral.com/1471-2474/15/17/prepub

## References

[B1] Imbens GW Rubin DB Bayesian inference for causal effects in randomized experiments with noncompliance Ann Stat 1997 25 1 305 327

[B2] Lamb SE Lall R Hansen Z Castelnuovo E Withers EJ Nichols V Griffiths F Potter R Szczepura A Underwood M A multicentred randomised controlled trial of a primary care-based cognitive behavioural programme for low back pain: the back skills training (BeST) trial Health Technol Assess 2010 14 41 1 253 20807469 10.3310/hta14410

[B3] Lamb SE Hansen Z Lall R Castelnuovo E Withers EJ Nichols V Potter R Underwood MR Group cognitive behavioural treatment for low-back pain in primary care: a randomised controlled trial and cost-effectiveness analysis Lancet 2010 375 9718 916 923 10.1016/S0140-6736(09)62164-4 20189241

[B4] Lamb SE Mistry D Lall R Hansen Z Evans D Withers EJ Underwood MR Group cognitive behavioural interventions for low back pain in primary care: extended follow-up of the back skills training trial (ISRCTN54717854) Pain 2012 153 494 501 10.1016/j.pain.2011.11.016 22226729

[B5] Bloom HS Accounting for no-shows in experimental evaluation designs Eval Rev 1984 8 2 225 246 10.1177/0193841X8400800205

[B6] Sommer A Zeger SL On estimating efficacy from clinical trials Stat Med 1991 10 1 45 52 10.1002/sim.4780100110 2006355

[B7] Angrist JD Imbens GW Rubin DB Identification of causal effects using instrumental variables J Am Stat Assoc 1996 91 434 444 455 10.1080/01621459.1996.10476902

[B8] Dunn G Maracy M Dowrick C Ayuso-Mateos JL Dalgard OS Page H Lehtinen V Casey P Wilkinson C Vazquez-Barquero JL Estimating psychological treatment effects from a randomised controlled trial with both non-compliance and loss to follow-up Br J Psychiatry 2003 183 4 323 331 10.1192/bjp.183.4.323 14519610

[B9] Skrondal A Rabe-Hesketh S Generalized latent variable modeling: multilevel, longitudinal, and structural equation models 2004 Boca Raton, FL: Chapman & Hall/CRC

[B10] Hansen Z Daykin A Lamb SE A cognitive-behavioural programme for the management of low back pain in primary care: a description and justification of the intervention used in the back skills training trial (BeST; ISRCTN 54717854) Physiotherapy 2010 96 2 87 94 10.1016/j.physio.2009.09.008 20420955

[B11] Lamb S Lall R Hansen Z Withers E Griffiths F Szczepura A Barlow J Underwood M Design considerations in a clinical trial of a cognitive behavioural intervention for the management of low back pain in primary care: back skills training trial BMC Musculoskelet Disord 2007 8 1 14 10.1186/1471-2474-8-14 17316434 PMC2147057

[B12] Burton K Waddell G The back book 2002 England: The Stationary Office Books

[B13] Roland M Morris R A study of the natural history of back pain: part I: development of a reliable and sensitive measure of disability in low-back pain Spine 1983 8 2 141 144 10.1097/00007632-198303000-00004 6222486

[B14] Underwood MR Barnett AG Vickers MR Evaluation of two time-specific back pain outcome measures Spine 1999 24 11 1104 1112 10.1097/00007632-199906010-00010 10361660

[B15] Dolan P Gudex C Kind P Williams A A social tariff for euroqol: results from a UK general population survey: centre for health economics 1995 York, UK: University of York

[B16] Imbens GW Rubin DB Estimating outcome distributions for compliers in instrumental variables models Rev Econ Stud 1997 64 4 555 574 10.2307/2971731

[B17] Muthén BO Beyond SEM: general latent variable modeling Behaviormetrika 2002 29 1; ISSU 51 81 118

[B18] StataCorp S Release 11 2009 StataCorp LP, College Station, TX: Statistical Software

[B19] Yau LHY Little RJ Inference for the complier-average causal effect from longitudinal data subject to noncompliance and missing data, with application to a job training assessment for the unemployed J Am Stat Assoc 2001 96 456 1232 1244 10.1198/016214501753381887

[B20] Hay E Mullis R Lewis M Vohora K Main C Watson P Dziedzic K Sim J Lowe CM Croft P Comparison of physical treatments versus a brief pain-management programme for back pain in primary care: a randomised clinical trial in physiotherapy practice Lancet 2005 365 9476 2024 2030 10.1016/S0140-6736(05)66696-2 15950716

[B21] Jo B Model misspecification sensitivity analysis in estimating causal effects of interventions with non-compliance Stat Med 2002 21 21 3161 3181 10.1002/sim.1267 12375297

[B22] Little RJ Yau LHY Statistical techniques for analyzing data from prevention trials: treatment of no-shows using Rubin's causal model Psychol Methods 1998 3 2 147 159

[B23] Mealli F Imbens GW Ferro S Biggeri A Analyzing a randomized trial on breast self-examination with noncompliance and missing outcomes Biostatistics 2004 5 2 207 222 10.1093/biostatistics/5.2.207 15054026

[B24] Dunn G Maracy M Tomenson B Estimating treatment effects from randomized clinical trials with noncompliance and loss to follow-up: the role of instrumental variable methods Stat Methods Med Res 2005 14 4 369 395 10.1191/0962280205sm403oa 16178138

[B25] Jo B Asparouhov T Muthén BO Intention-to-treat analysis in cluster randomized trials with noncompliance Stat Med 2008 27 27 5565 5577 10.1002/sim.3370 18623608 PMC2907896

[B26] Davies P Taylor F Beswick A Wise F Moxham T Rees K Ebrahim S Promoting patient uptake and adherence in cardiac rehabilitation Cochrane Database Syst Rev 2010 7 7 CD007131 20614453 10.1002/14651858.CD007131.pub2 PMC4164451

[B27] Heckman J Smith J Taber C Accounting for dropouts in evaluations of social programs Rev Econ Stat 1998 80 1 1 14 10.1162/003465398557203

